# Comparative efficacy of Chinese herbal injections for treating endometrial carcinoma: A Bayesian network meta-analysis

**DOI:** 10.1097/MD.0000000000034676

**Published:** 2023-10-13

**Authors:** Qin Li, Qi Chen, Zhi Zhang, Shuo Yang

**Affiliations:** a Institute of Information on Traditional Chinese Medicine, China Academy of Chinese Medical Sciences, Beijing, China; b School of Chinese Materia Medica, Beijing University of Chinese Medicine, Beijing, China.

**Keywords:** Bayesian model, Chinese herbal injections, endometrial carcinoma, network meta-analysis, randomized controlled trial

## Abstract

**Background:**

In view of the limitations of chemotherapy (CT) for endometrial carcinoma (EC) and the extensive use of Chinese herbal injections (CHIs), this network meta-analysis (NMA) compared the efficacy and safety of 6 CHIs combined with CT for EC.

**Methods:**

Several electronic databases were searched for randomized controlled trials (RCTs). The retrieval period was from the establishment of the databases to September 18, 2022. The quality of the literature was assessed after data extraction using Review Manager version 5.4. The Stata 13.1 and OpenBUGS3.2.3 software were used for data analysis. Cluster analysis was performed to compare the effect of CHIs between 2 different outcomes.

**Results:**

A total of 25 RCTs with 2023 patients were included. The findings demonstrated that when combined with CT, Aidi, Compound Kushen, Kangai, Eshuyou injection, and Shenmai injection can increase clinical efficacy compared to chemotherapy alone. The KPS level can be raised with aidi injection. Combining CT with injections of Aidi, Shenmai, Huangqi, and Compound Kushen can improve immunological performance. Combining CT with injections of Aidi, Huangqi, and Compound Kushen can lower serum amounts of tumor markers. Kangai injection was regarded as a good option for minimizing negative responses. According to the cluster analysis, the clinical effective rate, the KPS score, the level of CA125, and the clinical effective rate of Kangai injection and Aidi injection combined with CT were all better.

**Conclusion:**

Current evidence revealed that CHIs combined with CT have a better impact on patients with EC than CT alone. It’s possible that KangAi, Aidi, and Eshuyou infusion are the best CHIs for EC. Additionally, more high-quality RCTs are required in order to further corroborate the findings due to NMA’s limitations.

## 1. Introduction

A cancerous growth that develops in the endometrial epithelium is called endometrial carcinoma (EC). Recent years have seen a steady increase in the incidence of EC, and the average age of onset is getting younger and younger.^[1]^ The most prevalent malignant tumor of the female reproductive system in China and among industrialized countries is EC.^[2]^ There were 80,000 cases of EC in Chinese women in 2020, according to the most recent national cancer data published by the National Cancer Center in 2022. Most cases of EC develop after the age of 50, with the highest incidence occurring between the ages of 50 and 59. The symptoms of EC frequently include abnormal vaginal bleeding, discharge, uterine enlargement, and other advanced features. Radiotherapy and chemotherapy (CT) are frequently utilized as adjuvant therapies in the treatment of EC. For advanced, reproductive requirements and recurrent EC, the efficacy is constrained. Although CT treatment is effective, it frequently has major side effects, including menstruation abnormalities, liver and kidney damage, and bone marrow suppression.^[3]^ Studies have shown that EC can be caused by a variety of factors. The aging population, rising rates of metabolic disorders, infertility, and later marriage and childbearing ages are the main causes of the increase in morbidity and death.^[4]^

Chinese herbal injections (CHIs) are sterile preparations of a solution, emulsion, and powder or concentrated solution made into a solution before use for injection into a human body following the extraction and purification of Chinese medicinal materials in accordance with traditional Chinese medicine theory. In the adjuvant therapy of EC, CHIs may contribute to improvements in immune function, short-term efficacy, quality of life, adverse response reduction, tumor indicator decreasing, and other factors. Although CHIs are effective for the treatment of EC, prior research has focused solely at the efficacy and safety of combining a single CHI with chemotherapy. Since there have been no reported direct or indirect comparisons of the various CHIs used to treat EC, it is unclear which CHIs is the most successful one. Through network meta-analysis (NMA), this research seeks to implicitly compare the efficacy and safety of various CHIs combined with CT in the treatment of EC in the hope of offering some guidance for clinical treatment.

NMA is a modification of conventional meta-analysis. It expands upon the conventional direct meta-analysis of 2 groups of trials to compare and analyze a number of various treatment variables simultaneously. In order to provide trustworthy evidence for clinical practice, this research employed the NMA method to assess the effectiveness of various CHIs in the treatment of EC patients.

## 2. Methods

This study conforms to the PRISMA Extension Statement. The International Prospective Register of Systematic Reviews (PROSPERO) received the research protocol (CRD42022361515). Ethical approval from Ethics Committee and Institutional Review Board was not required, because this study does not involve human or animal testing, nor does it include case reports or a series of cases.

### 2.1. Inclusion criteria

The PICOS principles listed in the Cochrane Handbook served as the basis for the inclusion criteria for this investigation, which took into account patients, interventions, comparisons, outcome markers, and study design.

The PICOS guidelines provided in the Cochrane Handbook served as the foundation for the inclusion criteria of this study. The inclusion criteria were as follows: study type: randomized controlled trials (RCTs); study object: met the relevant diagnostic criteria for clinical EC; interventional measures: CHIs and Chemotherapy were administered to the experimental group, while Chemotherapy alone was administered to the control group; and outcome indicators: ① clinical effectiveness, ② levels of the T cell subsets (CD3+, CD4+, CD8+, and CD4+/CD8+),③ serum tumor markers (CA125 and HE4), ④ KarnoTsky scale, ⑤adverse reactions.

### 2.2. Exclusion criteria

The exclusion criteria were as follows: no full text; repeated published research; patients had any other primary tumors; interventions included other Chinese medicine treatments, such as acupuncture; and unavailability of outcome index data.

### 2.3. Retrieval strategy

The PubMed, Cochrane Library, Embase, China National Knowledge Infrastructure, VIP, and Wanfang databases were searched from their inception to September 18, 2022. Search strategies were constructed for 3 domains: EC; CHIs; and RCTs.

### 2.4. Data extraction

The literature was reviewed, and data were collected separately by 2 researchers. Endnote software was used to remove duplicate literature, read titles and abstracts, and screen out irrelevant studies. The remaining articles were read and rescreened according to the inclusion and exclusion criteria. Where there was disagreement, cases were discussed and resolved or referred to a third researcher for assistance with the judgment.

The extracted data included the following: basic information: title, first author’s name, and publication year; basic characteristics of the study subjects, including sample size and average age or age range; modes of intervention: dosage, frequency, and usage; key elements of bias risk assessment; and variation in the indicators before and after the intervention.

### 2.5. Quality assessment

The Cochrane Collaboration Bias Risk Assessment Tool was used to assess literature quality. The evaluation tool contains 7 elements: random sequence generation, allocation concealment, participant and staff blinding, outcome assessment blinding, incomplete outcomes, selective reporting, and other biases. Each bias has 3 levels: low risk, unclear risk, and high risk. If there were differences of opinion, they were resolved through discussion or with the assistance of a third party. Finally, a bias risk map was drawn.

### 2.6. Statistical analysis

Stata 13.1 software and OpenBUGS3.2.3 software were used for statistical analysis. Review Manager 5.3 was used to evaluate the quality of included RCT, OpenBUGS3.2.3 software was used for NMA, and Markov chain Monte Carlo method of random effect model was used for Bayesian inference. When calculating the effect size, the relative risk or odds ratio was used to represent the dichotomous data, and the weighted mean difference or standardized mean difference was used to represent the continuous data. The 95% confidence interval was set, and the random effect model was selected for data merging. OR exclusion 1 or MD value exclusion 0 was considered statistically significant. In OpenBUGS software, the number of simulated iterations is 200,000, with the first 20,000 iterations used for aging to eliminate the effect of initial values.

In addition, Stata 13.1 software was used to analyze the results and draw the evidence network diagram and cumulative probability ranking diagram of each outcome index. Visual analysis was performed on the relationship between different interventions. The point area represents the number of patients receiving relevant interventions, and the thickness of the line between each point represents the number of studies included. The efficacy of different interventions was ranked according to the area under the cumulative ranking curve (SUCRA). Cluster analysis was also performed to comprehensively compare the effect of CHIs on 2 different outcomes, with the interventions that were located in the upper-right corner being superior to the others. Draw a funnel plot to detect publication bias. In addition, if there is an intervention loop in the study, a consistency test is required.

## 3. Results

### 3.1. Characteristics of included studies

**Figure 1.**
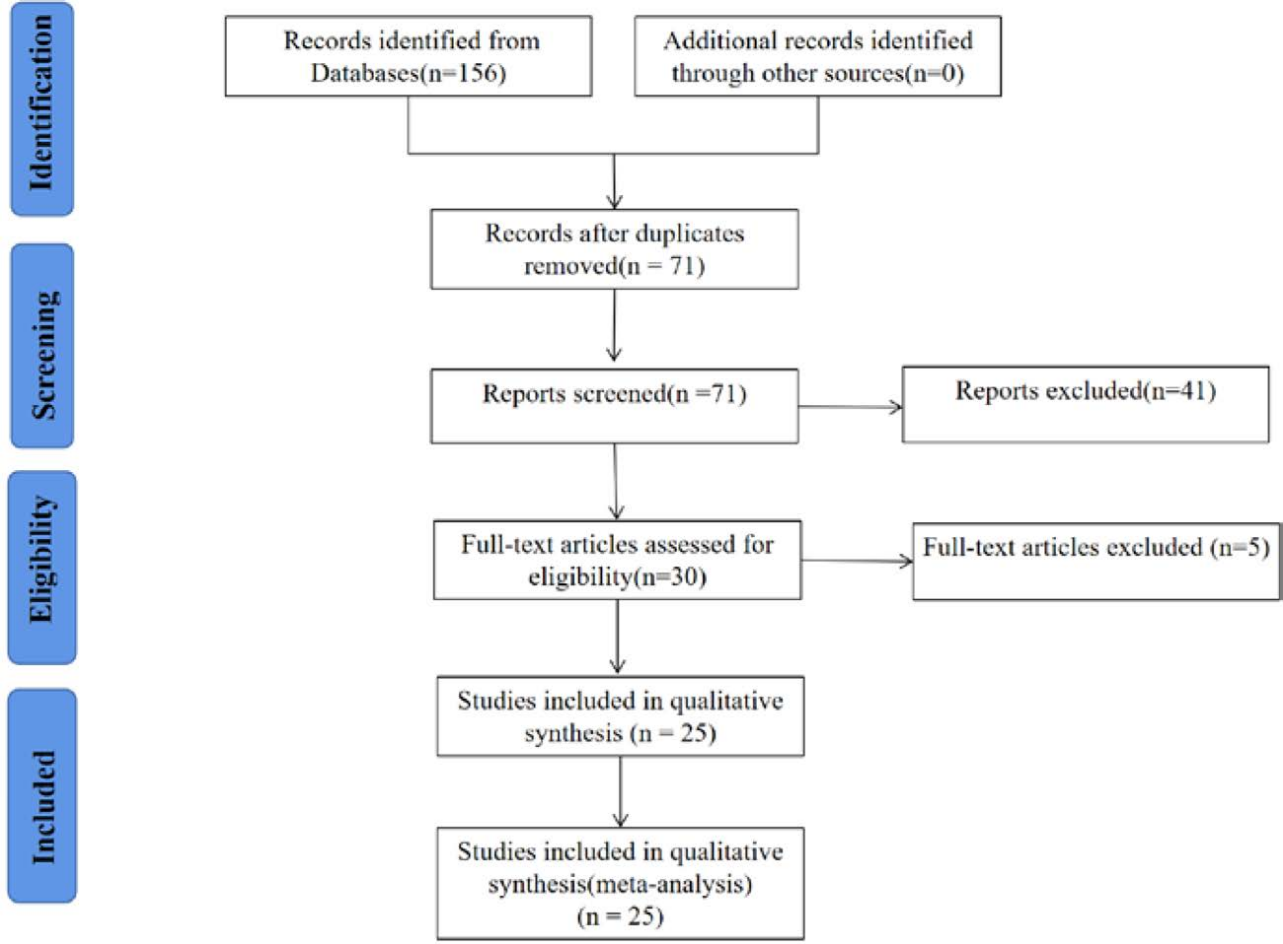
Flow chart of the search for eligible studies. n = number of articles.

A total of 156 related literature were obtained through database retrieval. After finding and eliminating 85 duplicate literature, 71 literature were included. After reading the title and abstract of the literature, 41 literatures were excluded. There were 2 literature that could not obtain the full text. Finally, a total of 25 studies^[5–29]^were analyzed involving 2023 participants. Literature screening process and results are shown in Figure 1.

**Figure 2.**
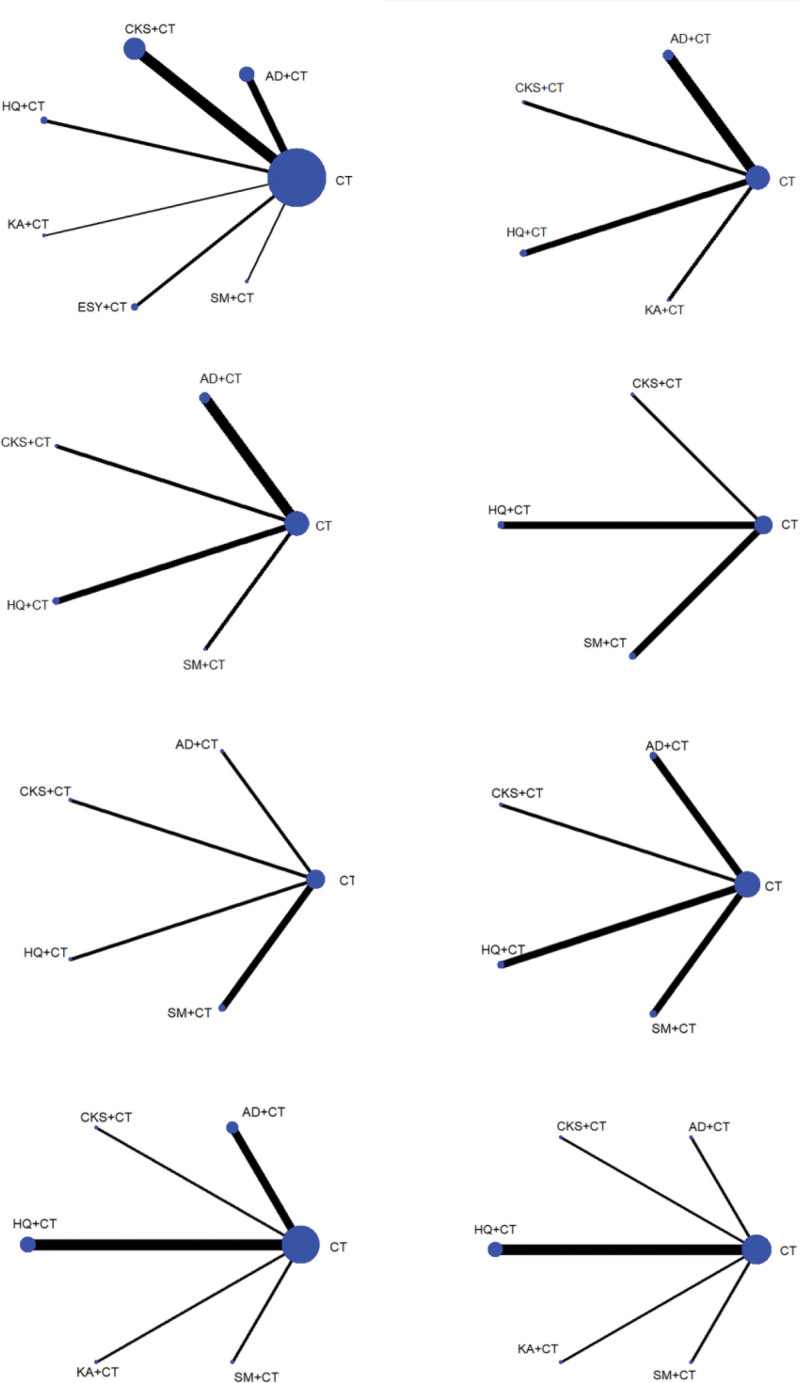
Network graph for different outcomes. (A) Clinical effective rate, (B) living conditions, (C) CD3+, (D) CD4+, (E) CD8+, (F) CD4+/CD8+, (G) HE4, (H) CA125. AD = Aidi injection, CKS = Compound Kushen injection, ESY = Eshuyou injection, HQ = Huangqi injection, KA = Kangai injection, SM = Shengmai injection.

All included studies were two-arm studies published between 2011 and 2021. For the convenience of description, AD is Aidi injection, CKS is Compound Kushen injection, HQ is Huangqi injection, KA is Kangai injection; ESY is Eshuyou injection, SM is Shenmai injection, CT is Chemotherapy. All the RCTs were two-arm studies involving 6 kinds of CHIs: AD (n = 5), CKS (n = 10), HQ (n = 5), KA (n = 1), SM (n = 2), ESY (n = 2). The control groups have been treated with CT. The treatment group was treated with CHIs on the basis of the control group. The details of the included CHIs are summarized in File S1, Supplemental Digital Content, http://links.lww.com/MD/J646, and the details of the selected studies are shown in Table 1. The network graphs of the interventions with different outcomes are depicted in Figure 2.

**Table 1:** The basic characteristics of the included studies.

Study	Sample size	Average age (yr)	Intervention		Course	Outcomes
			T	C		
Hu 2021^[5]^	30/30	C:43.6 ± 0.6 T:42.4 ± 0.1	Aidi injection + C	Chemotherapy + MegestrolCo Tablet 160 mg/d	3 mo	②③④⑤
Zhang 2021^[6]^	35/35	C:44.39 ± 3.28 T:43.68 ± 3.22	Aidi injection 50 mL + C	Chemotherapy + MegestrolCo Tablet 160 mg/d	3 mo	②③④⑤
Liu etal 2021^[7]^	43/43	≥18	Compound Kushen injection 20 mL + C	Chemotherapy + MegestrolCo Tablet 160 mg/d	4 wk	①②③④⑤
Wang et al 2021^[8]^	38/38	C:52.3 ± 8.6 T:52.3 ± 8.6	Huangqi injection 10 mL + C	Chemotherapy	63 d	①②③
Lou et al 2020^[9]^	55/55	C:58.05 ± 5.32 T:58.21 ± 5.12	Huangqi injection 250ml + C	Chemotherapy	3 wk	②③⑤
Ke and Ding 2019 ^[10]^	31/31	C:37.52 ± 4.83 T:39.14 ± 3.87	Huangqi injection + C	Chemotherapy	3 mo	④
Li and Si 2019 ^[11]^	50/50	C:32–64 T:31–63	Kangai injection 60ml + C	Chemotherapy	14 wk	①③④⑤
Zhao 2019 ^[12]^	25/25	C:50. 00 ± 5. 01 T:50. 00 ± 5. 00	Eshuyou injection 400 mg + C	Chemotherapy	4 wk	①④
Lu 2019^[13]^	39/39	C:40.2 ± 2.1 T:39.3 ± 2.3	Shenmai injection 100 mL + C	Chemotherapy	9 wk	②③
Huang 2018^[14]^	34/34	C:52.1 ± 7.0 T:51.4 ± 7.6	Aidi injection 50 mL + C	Chemotherapy + MegestrolCo Tablet 160 mg/d	6w	①②③④⑤
Fu 2016^[15]^	44/44	C:35.32 ± 2.61 T:35.36 ± 2.63	Compound Kushen injection 20 mL + C	Chemotherapy + MegestrolCo Tablet 160 mg/d	4 wk	①④⑤
Li 2017^[16]^	48/48	C: 36. 2 ± 3. 5 T:36.7 ± 3.1	Compound Kushen injection 20 mL + C	Chemotherapy + MegestrolCo Tablet 160 mg/d	4 wk	④⑤
Fu 2018^[17]^	32/32	C:31.21 ± 4.56 T:31.02 ± 4.26	Compound Kushen injection 20 mL + C	Chemotherapy + MegestrolCo Tablet 160 mg/d	4 wk	①③⑤
Chang et al 2016^[18]^	58/51	C:62.31 ± 10.72 T:63.00 ± 9.04	Huangqi injection 20 mL + C	Chemotherapy	2 mo	③④⑤
Gao 2018^[19]^	62/62	C:54.46 ± 7.39 T:55.91 ± 7.43	Huangqi injection 20 mL + C	Chemotherapy	4wk	①②③⑤
Yan 2017^[20]^	49/49	C:62.8 ± 4.1 T:63.1 ± 3.6	Compound kushen 20 mL + C	Chemotherapy	3 mo	①⑤
Li et al 2019^[21]^	25/25	C:44.8 ± 2.2 T:44.7 ± 2.2	Aidi injection 50 mL + C	Chemotherapy	/	①⑤
Jiang et al 2011^[22]^	30/28	C:40-66 T:34-68	Aidi injection 100 mL + C	Chemotherapy	10d	⑤
Li et al 2016^[23]^	50/50	C:47.6 ± 5.1 T:48.2 ± 3.7	Eshuyou injection 20 mL + C	Chemotherapy	/	①④⑤
Cui et al 2011^[24]^	22/20	C:41-46 T:42-66	Compound Kushen injection 20 mL + C	Chemotherapy	4 wk	①⑤
Liu 2011^[25]^	27/27	C:38--67 T:38--67	Compound Kushen injection 20 mL + C	Chemotherapy	2 wk	①⑤
Yang et al 2020^[26]^	30/30	C:53.44 ± 8.18 T:53.98 ± 7.59	Shenmai injection100 mL + C	Chemotherapy	9 wk	①②③⑤
Zhang et al 2019	60/60	C:35.8 ± 4.5 T:35.2 ± 4.4	Compound Kushen injection20 mL + C	Chemotherapy + MegestrolCo Tablet 160–320 mg/d	2 mo	①②
Yang et al 2020^[28]^	50/50	C:54.2 ± 4.5 T:55.1 ± 4.2	Compound Kushen injection20 mL + C	Chemotherapy + MegestrolCo Tablet 160 mg/d	4 wk	①②
Yang et al 2020^[29]^	50/50	C:54.5 ± 4.4 T:54.7 ± 4.6	Compound Kushen injection20 mL + C	Chemotherapy + MegestrolCo Tablet 160 mg/d	4 wk	①

① clinical effective rate; ② T cell subsets; ③ Serum tumor markers; ④ KPS score; ⑤ Adverse reactions.

C = control group, CT = chemotherapy, T = treatment group.

### 3.2. Bias assessment

In the selected RCTs, 9 RCTs used the random number table method to generate random sequences, which were rated as low risk; 3 RCTs were grouped according to treatment methods, and 1 RCT was divided according to the time of admission. These studies were rated as high risk. Other RCTs did not mention specific randomized grouping methods, and the risk of bias was unclear. None of the included studies mentioned allocation concealment and blinding information, and the risk of bias was unclear.

**Figure 3.**
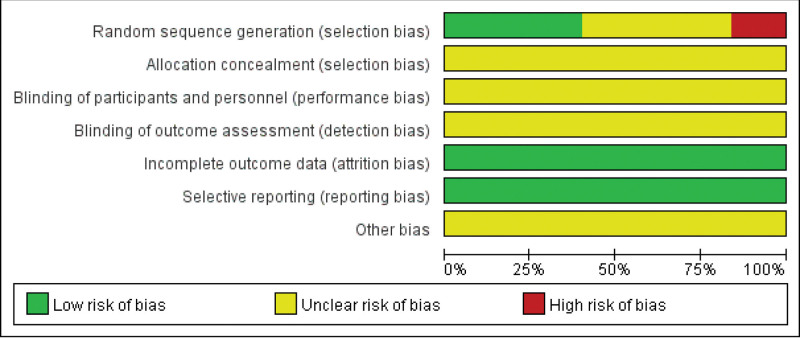
Risk of bias graph.

All studies have been regarded as low risk because they had no incomplete data. In terms of selective reporting, 25 RCTs reported outcome indicators that were expected to be measured, no early terminations were found, and selective reporting was low risk. Other bias risks were not described in detail in the included RCTs, and the risk of bias was unclear. The bias risk assessment details of all included studies are shown in Figure 3.

### 3.3. Outcome indicators

#### 3.3.1. Efficacy.

A total of 16 RCTs of 6 CHIs reported the clinical effective rate. ORs showed that comparing to using CT alone, combined with AD (OR = 4.26, 95% CI [2.32–7.85]), SM (OR = 0.47, 95% CI [0.27–0.93]), KA (OR = 7.37, 95% CI [1.99–27.32]), ESY (OR = 2.14, 95% CI [1.10–4.18]), SM (OR = 3.05, 95% CI [1.05–8.84]) can improve the clinical efficiency on the basis of the control group, and make the difference between the groups statistically significant. The specific values are shown in Table 2.

**Table 2:** Statistical results of network meta-analysis for the main outcomes (OR/MD value, 95% CI).

	Clinical effective rate	KPS score	CA125	HE4	CD3+	CD4+	CD8+	CD4+/CD8+
AD + CT vs								
CKS + CT	1.37 (0.65, 2.86)	0.93 (−18.2, 20.38)	−5.48 (−19, 7.98)	**21.82 (8.38, 35.03**)	**−14.41 (−25.85, −2.91**)	–	’−8.78 (−20.63, 3.01)	**0.99 (0.66, 1.49**)
HQ + CT	**2.44 (1.06, 5.60**)	0.81 (−18.11, 19.66)	**−10.83 (−21.72, −0.02**)	**20.84 (12.42, 28.9**)	**−14.13 (−23.06, −5.18**)	–	−5.59 (−17.18, 6.00)	1.12 (0.82, 1.55)
KA + CT	0.58 (0.14, 2.45)	2.69 (−23.36, 28.06)	−12.06 (−25.61, 1.56)	−9.76 (−25.45, 5.85)	–	–	–	–
ESY + CT	1.99 (0.80, 4.92)	–	–	–	–	–	–	–
SM + CT	1.40 (0.41, 4.76)	–	−3.87 (−17.39, 9.60)	**26.37 (15.03, 37.48**)	**−18.83 (−29.91, −7.75**)	–	−6.53 (−16.63, 3.59)	**0.94 (0.67, 1.32**)
CT	**4.26 (2.32, 7.85**)	**8.89 (3.29, 14.33**)	−3.84 (−13.46, 5.784)	**−34.02 (−39.96, −27.88**)	**23.28 (17.58, 28.95**)	–	6.79 (−1.46, 15.04)	**1.34 (1.06, 1.70**)
CKS + CT vs								
HQ + CT	1.78 (0.89, 3.59)	−0.12 (−26.07, 26.11)	−5.35 (−16.07, 5.28)	−0.98 (−14.16, 12.19)	0.28 (−11.83, 12.39)	−2.58 (−7.88, 2.73)	3.19 (−8.50, 14.92)	1.13 (0.76, 1.68)
KA + CT	0.42 (0.11, 1.67)	1.76 (−30.01, 32.88)	−6.59 (−20.05, 6.89)	**−31.59 (−50.24, −12.96**)	–	–	–	–
ESY + CT	1.45 (0.66, 3.20)	–	–	–	–	–	–	–
SM + CT	1.02 (0.33, 3.20)	–	1.60 (−11.8, 14.96)	4.54 (−10.57, 19.68)	−4.42 (−18.2, 9.34)	−2.74 (−8.14, 2.63)	2.26 (−7.98, 12.48)	0.95 (0.63, 1.43)
CT	**3.12 (2.05, 4.73**)	9.83 (−8.77, 28.65)	**−9.31 (−18.72, 0.13**)	**−12.2 (−24.06, −0.34**)	8.86 (−1.092, 18.81)	**8.81 (4.29, 13.33**)	−1.99 (−10.39, 6.41)	**1.35 (0.97, 1.88**)
HQ + CT vs								
KA + CT	0.24 (0.06, 0.99)	1.879 (−29.48, 31.9)	−1.23 (−12.06, 9.66)	**−30.61** (−46.09, −15.07)	–	–	–	–
ESY + CT	0.82 (0.34, 1.95)	–	–	–	–	–	–	–
SM + CT	0.57 (0.17, 1.91)	–	6.96 (−3.76, 17.73)	5.53 (−5.46, 16.52)	−4.71 (−16.43, 6.98)	−0.16 (−4.2, 3.88)	−0.94 (−10.97, 9.13)	**0.84 (0.61, 1.16**)
CT	1.75 (0.99, 3.07)	9.70 (−8.22, 27.81)	**−14.67 (−19.7, −9.667**)	**−13.18 (−18.82, −7.52**)	**9.15 (2.28, 16.00**)	**6.22 (3.47, 9.03**)	1.2 (−6.97, 9.30)	**1.19 (0.96, 1.47**)
KA + CT vs								
ESY + CT	3.44 (0.79, 14.97)	–	–	–	–	–	–	–
SM + CT	2.42 (0.45, 13.06)	–	8.19 (−5.32, 21.71)	**36.13 (18.9, 53.39**)	–	–	–	–
CT	**7.37 (1.99, 27.32**)	11.58 (−13.9, 36.56)	**−15.9 (−25.48, −6.29**)	**−43.79 (−58.27, −29.33**)	–	–	–	–
ESY + CT vs								
SM + CT	0.70 (0.20, 2.47)	–	–	–	–	–	–	–
CT	**2.14 (1.10, 4.18**)	–	–	–	–	–	0.26 (−5.60, 6.16)	–
SM + CT vs								
CT	**3.05 (1.05, 8.84**)		−7.71 (−17.23, 1.78)	−7.657 (−17.11, 1.78)	4.44 (−5.10, 13.91)	**6.06 (3.11, 9.00**)	–	**1.42 (1.11, 1.80**)

**Figure 4.**
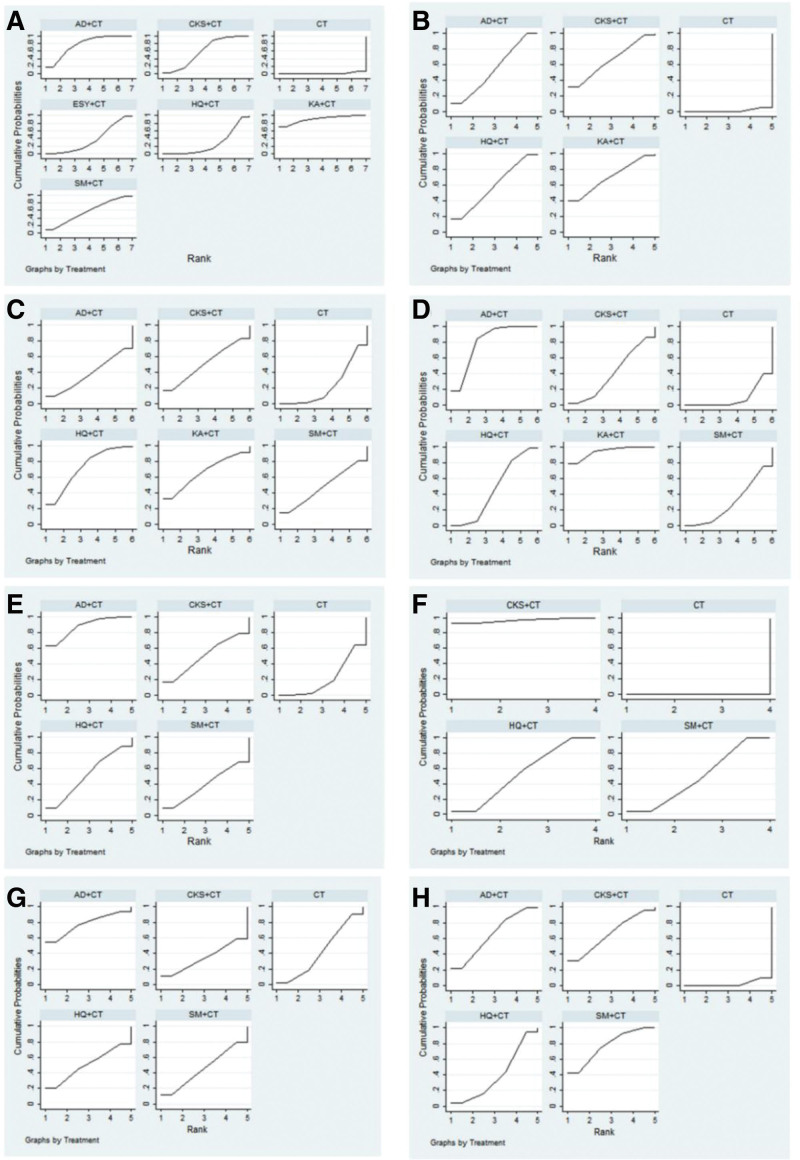
Plot of the surface under the cumulative ranking curves for all treatments. (A) Clinical effectiveness rate; (B) KPS score; (C) CA125; (D) HE4; (E) CD3+; (F) CD4+; (G) CD8+; (H) CD4+/CD8+. AD = Aidi injection, CKS = Compound Kushen injection, CT = chemotherapy, ESY = Eshuyou injection, HQ = Huangqi injection, KA = Kangai injection, SM = Shenmai injection.

After the ranking of each intervention efficacy, the combination of CT and KA (90.5%) had the highest probability of being the best treatment for EC in terms of improving the clinical effectiveness rate, followed by the combination of CT and AD (77.4%) and the combination of CT and CKS (59.8%). The ranking results of interventions are shown in Figure 4, and the SUCRA values are shown in Table 3.

**Table 3:** Surface under the cumulative ranking probabilities (SUCRA) results of main outcomes.

	Clinical effective rate		KPS score		CA125		HE4		CD3+		CD4+		CD8+		CD4+/CD8+	
	SUCRA (%)	RANK	SUCRA (%)	RANK	SUCRA (%)	RANK	SUCRA (%)	RANK	SUCRA (%)	RANK	SUCRA (%)	RANK	SUCRA (%)	RANK	SUCRA (%)	RANK
AD + CT	77.4	2	54	4	38.3	5	79.8	2	87.5	1	–	–	77.3	1	64.9	3
CKS + CT	59.8	3	65.4	2	51.3	3	40	4	50.3	3	96.9	1	34	5	65.4	2
HQ + CT	26.4	6	58.6	3	72.8	1	47.1	3	51.6	2	53.6	2	50.2	2	39.8	4
KA + CT	90.5	1	70.7	1	66.5	2	94.3	1	–	–	–	–	–	–	–	–
ESY + CT	37.3	5	–	–	–	–	–	–	–	–	–	–	–	–	–	–
SM + CT	57.6	4	–	–	48.1	4	29.8	5	39.4	4	49.5	3	45.7	3	77.2	1
CT	1	7	1.3	5	23	6	8.9	6	21.2	5	0	4	42.8	4	2.7	5

#### 3.3.2. T cell subsets.

The report of T Cell Subsets in this study mainly involved CD3+, CD4+, CD8+, and CD4+/CD8+. A total of 7 RCTs of 4 CHIs reported CD3+. Comparing to using CT alone, combined with AD (MD = 23.28, 95% CI [17.58–28.95]), HQ (MD = 9.15, 95% CI [2.28–16.00]), on the basis of chemotherapy can improve CD3 + decline in patients and make the difference between groups statistically significant. A total of 5 RCTs of 3 CHIs reported CD4+. Comparing to using CT alone, combined with CKS (MD = 8.81, 95% CI [4.29–13.33]), HQ (MD = 6.22, 95% CI [3.47–9.03]), and SM (MD = 6.06, 95% CI [3.11–9.00]), on the basis of chemotherapy can improve CD4 + decline in patients and make the difference between groups statistically significant. A total of 5 RCTs of 4 CHIs reported CD8+. A total of 7 RCTs of 4 CHIs reported CD4+/CD8+. Comparing to using CT alone, combined with AD (MD = 1.34, 95% CI [1.06–1.70]), HQ (MD = 1.19, 95% CI [0.96–1.47]), CKS (MD = 1.35, 95% CI [0.97–1.88]), on the basis of chemotherapy can improve CD4+/CD8+ decline in patients and make the difference between groups statistically significant. In terms of improving the decrease of CD8 + in patients, there was no difference between the combined AD\CKS\HQ\ESY groups on the basis of chemotherapy compared with chemotherapy alone. The specific values are shown in Table 2.

After the ranking of each intervention efficacy, the combination of CT and AD (87.5%) had the highest probability of improving CD3 + decline in patients. The combination of CT and CKS (77.3%) was the best treatment option in reducing CD8 + decline. In terms of reducing the decrease of CD4 + and CD8+, the combination of CT and SM (77.2%) was the best treatment for patients, followed by the combination of CT and CKS (65.4%) and the combination of CT and AD (64.9%). The ranking results of interventions are shown in Figure 4, and the SUCRA values are shown in Table 3.

#### 3.3.3. Serum tumor markers.

The serum tumor markers in this study mainly detected the changes of HE4 and CA125. A total of 10 RCTs mentioned HE4, involving 5 CHIs. Comparing to using CT alone, combined with AD (MD = −34.02, 95% CI [−39.96, −27.88]), CKS (MD = −12.2, 95% CI [−24.06, −0.34]), HQ (MD = −13.18, 95% CI [−18.82, −7.52]), KA (MD = − 43.79, 95% CI [−58.27, −29.33]) on the basis of chemotherapy can reduce HE4, and make the difference between groups statistically significant. A total of 8 RCTs mentioned CA125, involving 5 CHIs. Comparing to using CT alone, combined with CKS (MD = −9.31, 95% CI [−18.72, 0.13]), HQ (MD = −14.67, 95% CI [−19.7, −9.66]), KA (MD = −15.9, 95% CI [−25.48, −6.294]), on the basis of chemotherapy can reduce CA125, and make the difference between groups statistically significant. The specific values are shown in Table 2.

After the ranking of each intervention efficacy, the combination of CT and KA (94.3%) had the highest probability of reducing HE4 in patients, followed by the combination of CT and AD (79.8%). The combination of CT and HQ (72.8%) was the best treatment option for reducing CA125, followed by the combination of CT and KA (66.5 %). The ranking results of interventions are shown in Figure 4, and the SUCRA values are shown in Table 3.

#### 3.3.4. KPS score.

There were 11 RCTs involving Karnofsky performance score (KPS) score of quality of life, involving 5 CHIs and 6 interventions. The results showed that comparing to CT alone, the combined with AD (MD = 8.89, 95% CI [3.29,14.33]) on the basis of chemotherapy could improve the quality of life, and the difference between the groups was statistically significant. The specific values are shown in Table 2.

After the ranking of each intervention efficacy, the combination of CT and KA (70.7%) had the highest probability of improving the quality of life of patients, followed by the combination of CT and CKS (64.4%) and HQ (58.6%). The ranking results of interventions are shown in Figure 4, and the SUCRA values are shown in Table 3.

#### 3.3.5. Adverse reactions.

A total of 19 RCTs had adverse reactions during the study. The adverse reactions mainly included 5 aspects: leukopenia, liver and kidney dysfunction, myelosuppression, gastrointestinal reactions and thrombocytopenia.

A total of 12 RCTs reported leukopenia, involving 5 CHIs. NMA showed that there were significant differences between CT and AD (OR = 0.24, 95% CI [0.09, 0.62]), CKS (OR = 0.31, 95% CI [0.18, 0.53]), KA (OR = 0.10, 95% CI [0.03, 0.38]) and CT alone. HQ combined with CT was better than KA combined with CT (OR = 7.10, 95% CI [1.48, 34.16]) in the respect of relieving leukopenia (Table 4).

**Table 4:** Statistical results of network meta-analysis for ADR outcomes (OR value, 95% CI).

	Leukopenia	Liver and kidney dysfunction	Myelosuppression	Gastrointestinal reactions	Thrombocytopenia
AD + CT vs					
CKS + CT	0.78 (0.26, 2.33)	**2.40 (1.07, 5.42**)	2.55 (0.93, 7.01)	2.07 (0.93, 4.60)	1.72 (0.64, 4.61)
HQ + CT	0.32 (0.09, 1.18)	**3.22 (1.62, 6.40**)	**3.49 (1.69, 7.20**)	**2.93 (1.69, 5.07**)	**3.07 (1.72, 5.49**)
KA + CT	2.29 (0.46, 11.46)	2.19 (0.90, 5.31)	2.10 (0.99, 4.48)	**2.29 (1.21, 4.32**)	1.58 (0.53, 4.76)
ESY + CT	–	–	1.36 (0.29, 6.43)	**4.47 (1.75, 11.43**)	1.28 (0.32, 5.07)
CT	**0.24 (0.09, 0.62**)	2.55 (0.62, 10.49)	**2.89 (1.07, 7.82**)	0.77 (0.34, 1.75)	1.17 (0.39, 3.51)
CKS + CT vs					
HQ + CT	0.41 (0.15, 1.17)	0.94 (0.18, 4.82)	0.88 (0.21, 3.64)	0.78 (0.34, 1.81)	1.47 (0.34, 6.44)
KA + CT	2.94 (0.72, 12.01)	1.26 (0.26, 6.08)	1.21 (0.35, 4.14)	1.53 (0.52, 4.54)	2.63 (0.76, 9.11)
ESY + CT	–	–	0.73 (0.21, 2.54)	0.26 (0.10, 0.71)	1.35 (0.29, 6.41)
CT	**0.31 (0.18, 0.53**)	0.86 (0.16, 4.56)	0.47 (0.07, 2.98)	**0.34 (0.20, 0.59**)	1.09 (0.19, 6.36)
HQ + CT vs					
KA + CT	**7.10 (1.48, 34.16**)	1.10 (0.33, 3.66)	1.87 (0.29, 11.92)	1.96 (0.63, 6.07)	1.35 (0.25, 7.32)
ESY + CT	–	–	2.56 (0.46, 14.20)	0.34 (0.12, 0.95)	2.40 (0.54, 10.72)
CT	0.74 (0.31, 1.79)	1.47 (0.48, 4.52)	1.54 (0.27, 8.68)	**0.44 (0.23, 0.83**)	**1.24 (1.21, 7.23**)
KA + CT vs					
ESY + CT	–	–	1.21 (0.34, 4.28)	0.17 (0.05, 0.60)	1.09 (0.25, 4.75)
CT	**0.10 (0.03, 0.38**)	0.75 (0.26, 2.17)	1.66 (0.58, 4.73)	**0.22 (0.09, 0.57**)	1.94 (0.56, 6.72)
ESY + CT vs					
CT	–	–	0.73 (0.21, 2.53)	0.71 (0.27, 1.86)	0.56 (0.18, 1.76)

A total of 12 RCTs reported liver and kidney dysfunction, involving 4 CHIs. There was no statistically significant difference between the interventions.

A total of 12 RCTs reported myelosuppression, involving 5 CHIs. NMA showed that CT and AD (OR = 2.89, 95% CI [1.07, 7.82]), compared with CT alone, the difference was statistically significant (Table 4).

A total of 13 RCTs reported gastrointestinal reactions, involving 5 CHIs. NMA showed that CT and KA (OR = 0.34, 95% CI [0.20, 0.59]), HQ (OR = 0.44, 95% CI [0.23, 0.83]), KA (OR = 0.22, 95% CI [0.09, 0.57]) were significantly different from CT alone (Table 4).

A total of 13 RCTs reported thrombocytopenia, involving 5 CHIs. NMA showed that CT and HQ (OR = 1.24, 95% CI [1.21, 7.23]) were significantly different from CT alone (Table 4).

The results of area under the adverse reaction curve showed that, that ESY combined with CT was the best treatment for improving gastrointestinal reactions (93.2%), thrombocytopenia (80.3%) and myelosuppression (91.9%) in EC patients, KAI combined with CT was more effective in reducing leukopenia (93.5%) and improving liver and kidney dysfunction (96.3%). The details are shown in Table 5.

**Table 5:** Surface under the cumulative ranking probabilities (SUCRA) results of ADR outcomes.

	Leukopenia		Liver and kidney dysfunction		Myelosuppression		Gastrointestinal reactions		Thrombocytopenia	
	SUCRA (%)	RANK	SUCRA (%)	RANK	SUCRA (%)	RANK	SUCRA (%)	RANK	SUCRA (%)	RANK
AD + CT	41.4	3	25.9	5	22.7	6	46.1	3	14.8	6
CKS + CT	79	2	50.2	2	52.3	3	26.1	5	50.8	4
HQ + CT	5.6	5	42	3	69.2	2	40.8	4	62	3
KA + CT	93.5	1	96.3	1	34.6	4	84.6	2	67.4	2
ESY + CT	–	–	–	–	91.9	1	93.2	1	80.3	1
CT	30.7	4	35.5	4	29.2	5	9.3	6	24.6	5

Other adverse reactions involved were reported as follows. In Huang’s study,^[14]^ 5 patients in the treatment group had alopecia, while 24 in the control group. There were 3 cases of cardiac dysfunction in the treatment group and 10 cases in the control group. In Chang’s study,^[18]^ HQ combined with CT as an intervention in the treatment group, the treatment group had 23 cases of fever, 30 cases in the control group. There were 18 cases of vomiting in the treatment group and 26 cases in the control group. Treatment group had 24 diarrhea constipation, 31 cases in the control group. In Gao’s study^[19]^ HQ combined with CT as the intervention of the treatment group, 5 cases had a fever in the treatment group and 8 cases in the control group. In Yan’s study,^[20]^ CKS combined with CT as the intervention of the treatment group, there was 1 case of oral mucosal erosion in the treatment group and 9 cases in the control group. There were 8 cases of hair loss in treatment group and 20 cases in control group. In Li’s study^,[23]^ ESY combined with CT as the intervention of the treatment group. There were 7 cases of hemoglobin reduction in the treatment group and 6 cases in the control group. There were 16 cases of nausea and vomiting in the treatment group and 19 cases in the control group. There were 37 cases of alopecia in the treatment group and 41 cases in the control group. In Cui’s study,^[24]^ AD combined with CT as the intervention of the treatment group, there were 5 cases of oral mucositis in the treatment group and 6 cases in the control group. In Liu’s study,^[7]^ the patients in the observation group and the control group had symptoms such as nausea, vomiting, thrombocytopenia and leukopenia during the treatment. After 2 months of withdrawal after the whole course of treatment, the symptoms gradually relieved or disappeared, and the specific number was not yet clear. In Zhang’s study,^[27]^ during the treatment, there were 3 cases of adverse reactions in the treatment group and 8 cases in the control group. The incidence of adverse reactions was 5% and 13.33% respectively.

### 3.4. Cluster analysis

**Figure 5.**
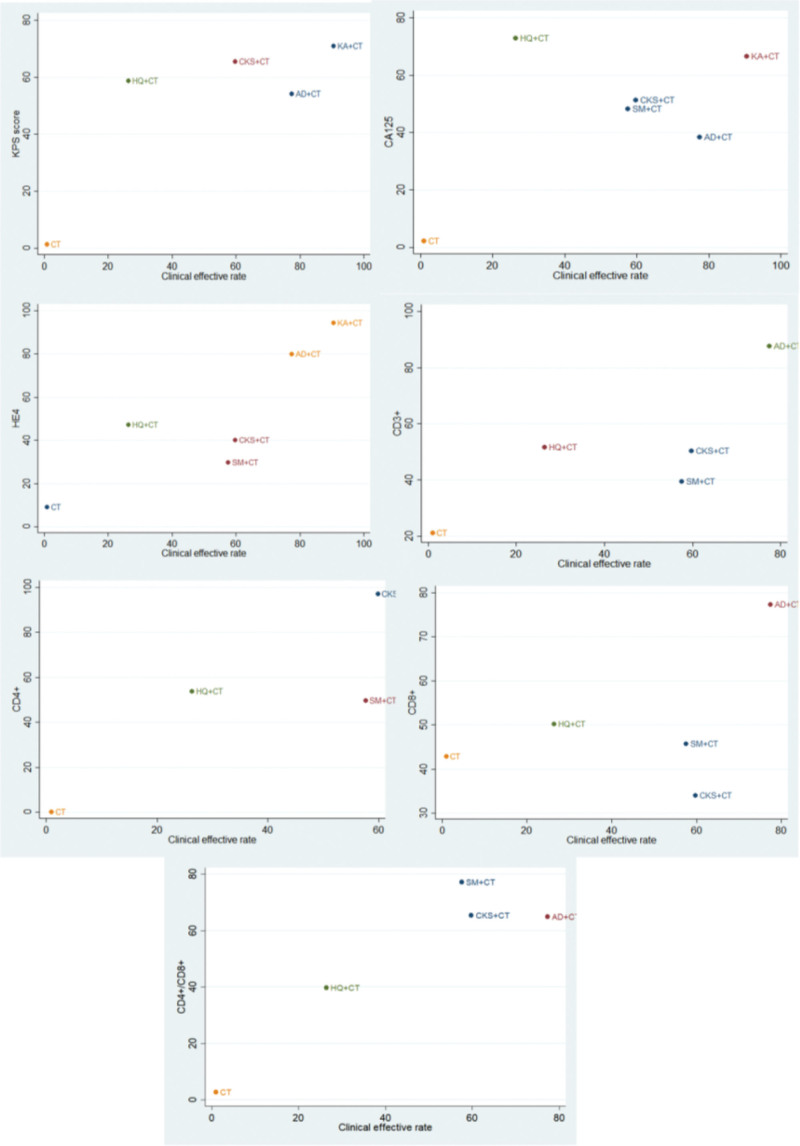
Cluster analysis plot for outcomes. Interventions located in the upper right corner indicate optimal therapy for 2 different outcomes.

The cluster analysis was carried out to integrate the clinical effective rate with each of the first 7 secondary outcomes based on the SUCRA of the interventions shared by the paired outcomes. As shown in Figure 5, the clinical effective rate and level of CA125, KA, and AD combined with CT were both superior in terms of the KPS score and clinical effective rate. KA and AD were the optimal therapies in terms of the clinically effective rate and level of HE4. Clinical effectiveness and CD3 + and CD4+/CD8 + levels showed that AD combined with CT was more favorable. CKS combined with CT was preferred in terms of clinical effective rate and CD4 + level.

### 3.5. Publication bias

**Figure 6.**
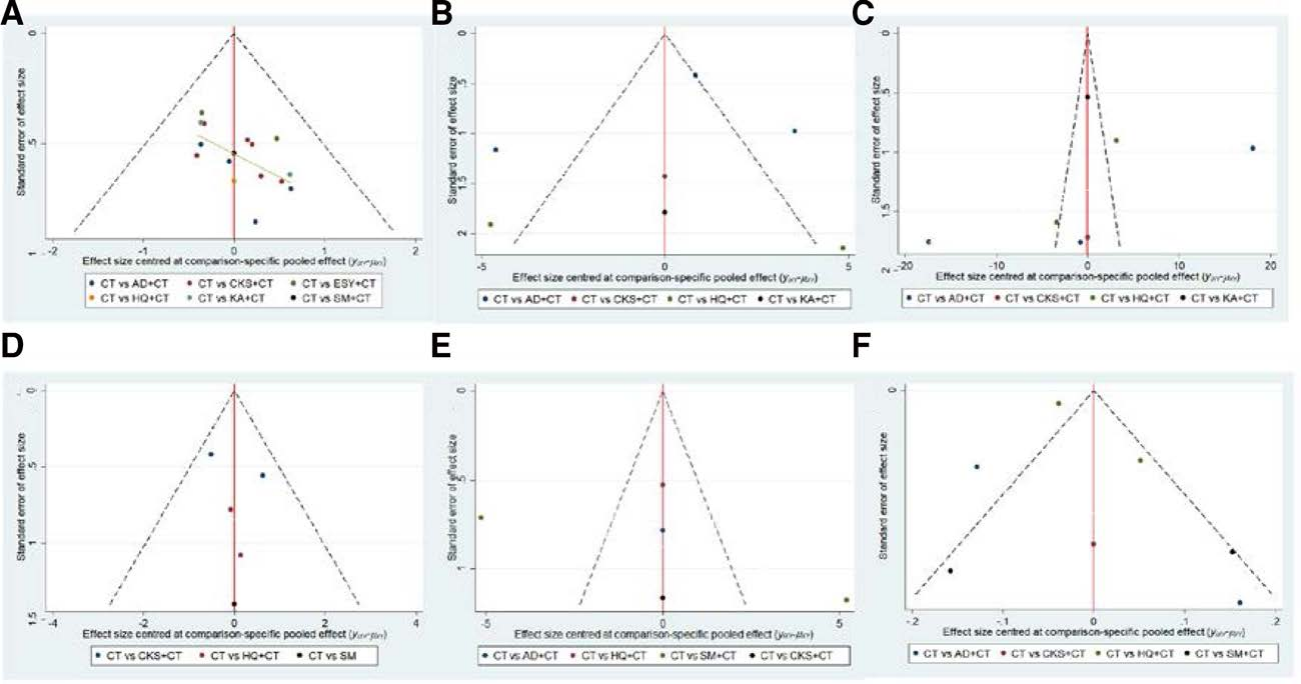
Funenel plot. (A) Clinical effective rate, (B) living conditions, (C) CD3+, (D) CD4+, (E) CD8+, (F) CD4+/CD8+. AD = Aidi injection, CKS = Compound Kushen injection, CT = chemotherapy, ESY = Eshuyou injection, HQ = Huangqi injection, KA = Kangai injection, SM = Shenmai injection.

This study used funnel plots to illustrate the outcome indicators. As shown in Figure 6, the clinical effective rate funnel plots were not perfectly symmetrical, indicating a potential publication bias in this study that may have been brought on by the small sample effect. Contrarily, the funnel charts indicated no publication bias because they displayed unimpressive asymmetry on both sides of the centerline in the living situations (CD3+, CD4+, CD8+, and CD4+/CD8+).

### 3.6. GRADE quality of evidence

The quality of the evidence for the study’s outcome indicators was assessed using the GRADE grading system in network meta-analysis.^[30]^ The evaluation largely took into account 5 degrading factors: publication bias, indirection, inconsistency, and bias risk. The comparison between interventions used evidence of low or very low quality, according to GRADE classification criteria. The heterogeneity and sample size of the included research results affect consistency and accuracy, respectively, and because the majority of the literature does not mention that blinding and allocation concealment can increase the risk of bias, this results in a low overall evaluation of the quality of the evidence (Table 6).

**Table 6:** Detailed information about the GRADE Quality of Evidence.

Outcomes	Risk of bias	Inconsistency	Indirectness	Imprecision	Publication Bias	GRADE evidence quality
Clinical efficacy	High risk of bias	No inconsistency	Serious indirectness	No imprecision	No publication bias	Low quality
T cell subsets	High risk of bias	No inconsistency	Serious indirectness	No imprecision	No publication bias	Very low quality
Serum tumor markers	High risk of bias	No inconsistency	Serious indirectness	No imprecision	No publication bias	Very low quality
Kps score	High risk of bias	No inconsistency	Serious indirectness	No imprecision	No publication bias	Very low quality
Adverse actions	High risk of bias	No inconsistency	Serious indirectness	No imprecision	No publication bias	Low quality

## 4. Discussion

Based on the NMA method, this study thoroughly assessed the efficacy and safety of 6 CHIs combined with CT in the treatment of EC using the NMA approach. Due to the NMA’s non-closed loops, the assumption of consistency between direct and indirect evidence was not used. In this network meta-analysis, the clinical effective rate, KPS score, T cell subsets (CD3+, CD4+, CD4+/CD8+), serum tumor markers (CA125, HE4), and adverse reactions were all shown to be 5 interesting outcomes. According to the findings, the combination of CT with AD, CKS, KA, ESY, and SM had a better outcome than CT alone in terms of clinical effective rate.

This study thoroughly assessed the efficacy and safety of 6 CHIs combined with CT in the treatment of EC using the NMA approach. Due to the NMA’s non-closed loops, the assumption of consistency between direct and indirect evidence was not used. In this network meta-analysis, the clinical effective rate, KPS score, T cell subsets (CD3+, CD4+, CD4+/CD8+), serum tumor markers (CA125, HE4), and adverse events were all shown to be 5 interesting outcomes. According to the findings, the combination of CT with AD, CKS, KA, ESY, and SM had a better outcome than CT alone in terms of clinical effective rate.

Based on SUCRA values, KA combined with CT ranked the highest in improving the clinical effective rate, KPS score and HE4. What is more, AD combined with CT ranked second in the clinical effective rate but ranked the highest in the level of CD3 + and CD8+. According to the cluster analysis, KA and AD combined with CT were similarly superior in terms of the clinical effective rate and the KPS score, the clinical effective rate and the level of CA125. KA and AD with CT were the best therapies in terms of the clinically effective rate and the level of HE4. Thus, EC should pay closer attention to KA, AD, and CT when they are paired with CT. The level of support for this finding could be weaker, though, given the small sample size.

As for safety, 19 RCTs reported adverse reactions. In terms of reduction in leukopenia, liver and kidney dysfunction, KA combined with CT performed significantly better than the other. KA combined with CT had the best effect on reducing gastrointestinal reactions, myelosuppression and thrombocytopenia. However, clinicians should choose different treatments based on patients’ specific conditions.

There is no name of EC in traditional Chinese medicine. EC vaginal hemorrhage and drainage are characterized as “Zhengjia,” “Daixia,” “Benglou,” and so on based on their symptoms. This condition may be brought on by internal harm to the 7 emotions, mental depression, liver dysfunction, qi stagnation, and blood stasis; or it may result from improper diet (clean), spleen and stomach disorders, phlegmy wet suisheng, and endogenous blood stasis; qi and blood stasis, meridian congestion, stagnation of fire, accumulated dampness into heat, gathered in the lower coke, damp heat, and blood stasis.^[31]^ CHIs has the impact of strengthening the body’s capacity to fight cancer, controlling intrinsic functions, and improving the internal environment of the human body.

Pharmacologic investigations have demonstrated that AD can prevent tumor growth, directly limit cancer cell proliferation, trigger cancer cell apoptosis, and keep platelets and white blood cell counts reasonably steady.^[32]^ The primary ingredient in CKS is a marine extract, which has can the capacity to inhibit the growth of tumor cells.^[33]^ On a certain level, CKS slows the proliferation of malignant cells. Its principal device can prevent tumor cell invasion.^[33]^

In addition to other pharmacological actions, HQ has a powerful heart, vasodilator, liver, and myocardial protection, improves immune function, and increased white blood cells.^[34]^ After Huang Qi injection was applied to HEC-1-B cells, it could significantly reduce the protein content and mRNA expression level of TGF-β1, and then inhibit the proliferation and invasion of cells.^[35]^ Ouyang obtained the same results as Qiu after intervening HEC-1-B cells with Huang Qi injection injection.^[36]^ Ginseng, astragalus, and matrine are the 3 major ingredients of KA. Vascular endothelial cells and tumors can both be inhibited by matrine. Interleukin-6, macrophages, and bone marrow stromal cells can all be stimulated by ginsenosides. The immune system is regulated and safeguarded by astragalus.^[37]^ Research has revealed that ESY injection can have a greater anti-tumor effect, decrease the proportion of cells in the cell cycle division phase, and decrease the proliferation rate of human endometrial cancer cells.^[38]^ Ginsenosides, ginseng polysaccharides, steroidal glycosides, organic acids, and other substances make up the majority of SM. These powerful ingredients can enhance the body’s organ anti-stress capacity and control and enhance immunological function.^[39]^

At the same time, this study also has some limitations. First, all patients were Chinese, and all investigations were carried out in China. As a result, the results might not be generalizable. Secondly, the results could not be sufficiently convincing due to the small number of RCTs included in the research. Finally, the quality of evidence for most outcomes was low and need further evidence.

## 5. Conclusion

In conclusion, current research has shown that CHIs and CT work better together to treat patients with EC than CT alone. Also, more high-quality RCTs are required in order to further confirm the findings due to NMA’s limitations.

## Author contributions

**Data curation:** Qin Li, Zhi Zhang.

**Formal analysis:** Qi Chen.

**Methodology:** Shuo Yang.

**Software:** Qin Li, Qi Chen, Zhi Zhang.

**Writing – original draft:** Qin Li.

**Writing – review & editing:** Shuo Yang.

## Supplementary Material
