# Supplementary material for: Comparative efficacy of Chinese herbal injections for treating endometrial carcinoma: A Bayesian network meta-analysis
Source: Medicine (Baltimore). 2023 Oct 13;102(41):e34676. doi: 10.1097/MD.0000000000034676 (PMC10578732; doi:10.1097/MD.0000000000034676)
Supplement: Supplementary file 1 [file medi-102-e34676-s001.docx]

**File 1: Detailed information about the CHIs used in the included studies**

| Study | Chinese herbal injection | Source | Species | Quality control reported? (Y/N) | Chemical analysis reported? (Y/N) |
| --- | --- | --- | --- | --- | --- |
| Hu2021 | Aidi injection | Guizhou Yibai Pharmaceutical Co., Ltd. | Mylabris 1.5g (animal drug), Ginseng Radix Et Rhizoma 50g, Astmgali Radix 100g, Acanthopanacis Senticosi Radix Et Rhizoma Seu Caulis 150g, Astmgali Radix 20g | Y-National Pharmaceutical Standard Z52020236; | N |
| Zhang 2021 | Aidi injection | Not mentioned | Mylabris 1.5g (animal drug), Ginseng Radix Et Rhizoma 50g, Astmgali Radix 100g, Acanthopanacis Senticosi Radix Et Rhizoma Seu Caulis 150g, Astmgali Radix 20g | Y-National Pharmaceutical Standard Z52020236; | N |
| Liu etal2021 | Compound kushen injection | Shanxi Zhendong Pharmaceutical Co., Ltd. | Radix Sophorae Flavescentis 1400g, Heterosmilacis Rhizoma 600g | N | N |
| Wang etal.2021 | Huangqi injection | Shenwei Pharmaceutical Co., Ltd. | Coicis Semen oil | Y-National Pharmaceutical Standard Z13020999 | N |
| Lou etal.2020 | Huangqi injection | Not mentioned | Coicis Semen oil | N | N |
| Ke and Ding 2019 | Huangqi injection | Not mentioned | Coicis Semen oil | N | N |
| Li and Si 2019 | Kangai injection | Changbaishan Pharmaceutical Co., Ltd. | Astragali Radix 300g, Ginseng Radix Et Rhizoma 100g, Matrine 10g (Chemical medicine) | Y-National Pharmaceutical Standard Z20026868 | N |
| Zhao 2019 | Eshuyou injection | Not mentioned | Codonopsis Radix, Astmgali Radix | N | N |
| Lu2019 | Shenmai injection | Not mentioned | Red Ginseng, Radix Ophiopogonis | N | N |
| Huang2018 | Aidi injection | Guizhou Yibai Pharmaceutical Co., Ltd. | Mylabris 1.5g (animal drug), Ginseng Radix Et Rhizoma 50g, Astmgali Radix 100g, Acanthopanacis Senticosi Radix Et Rhizoma Seu Caulis 150g, Astmgali Radix 20g | China lot number: 20150905 and 20161203 | N |
| Fu 2016 | Compound kushen injection | Not mentioned | 4Radix Sophorae Flavescentis 1400g, Heterosmilacis Rhizoma 600g | N | N |
| Li2017 | Compound kushen injection | Shanxi Zhendong Pharmaceutical Co., Ltd. | Radix Sophorae Flavescentis 1400g, Heterosmilacis Rhizoma 600g | Y-National Pharmaceutical Standard Z14021230 | N |
| Fu2018 | Compound kushen injection | Shanxi Zhendong Pharmaceutical Co., Ltd. | Radix Sophorae Flavescentis 1400g, Heterosmilacis Rhizoma 600g | Y-National Pharmaceutical Standard Z14021231 | N |
| Chang etal.2016 | Huangqi injection | Shenwei Pharmaceutical Co., Ltd. | Coicis Semen oil | China lot number: 20140201 | N |
| Gao2018 | Huangqi injection | Not mentioned | Coicis Semen oil | 1. National Pharmaceutical Standard   Z23020781 | N |
| Yan2017 | Compound kushen | Not mentioned | Radix Sophorae Flavescentis 1400g, Heterosmilacis Rhizoma 600g | N | N |
| Li etal.2019 | Aidi injection | Not mentioned | Mylabris 1.5g (animal drug), Ginseng Radix Et Rhizoma 50g, Astmgali Radix 100g, Acanthopanacis Senticosi Radix Et Rhizoma Seu Caulis 150g, Astmgali Radix 20g | N | N |
| Jiang etal.2011 | Aidi injection | Not mentioned | Mylabris 1.5g (animal drug), Ginseng Radix Et Rhizoma 50g, Astmgali Radix 100g, Acanthopanacis Senticosi Radix Et Rhizoma Seu Caulis 150g, Astmgali Radix 20g | Y-National Pharmaceutical Standard Z52020236 | N |
| Li etal.2016 | Eshuyou injection | Zhejiang Tianrui Pharmaceutical Co., Ltd.; | /Codonopsis Radix, Astmgali Radix | Y-National Pharmaceutical Standard H33022270 and H20064312 | N |
| Cui etal.2011 | Compound kushen | Not mentioned | Radix Sophorae Flavescentis 1400g, Heterosmilacis Rhizoma 600g | N | N |
| Liu 2011 | Compound kushen | Not mentioned | Radix Sophorae Flavescentis 1400g, Heterosmilacis Rhizoma 600g | N | N |
| Yang etal.2020 | Shenmai injection | Zhengda Qingchunbao Pharmaceutical Co., Ltd.; | Red Ginseng, Radix Ophiopogonis | Y-National Pharmaceutical Standard Z33020019 | N |
| Zhang et al.2019 | Compound kushen injection | Not mentioned | Radix Sophorae Flavescentis 1400g, Heterosmilacis Rhizoma 600g | N | N |
| Yang etal.2020 | Compound kushen injection | Shanxi Zhendong Pharmaceutical Co., Ltd. | Radix Sophorae Flavescentis 1400g, Heterosmilacis Rhizoma 600g | Y-National Pharmaceutical Standard Z14021231 | N |
| Yang et al.2020 | Compound kushen injection | Shanxi Zhendong Pharmaceutical Co., Ltd. | Radix Sophorae Flavescentis 1400g, Heterosmilacis Rhizoma 600g | Y-National Pharmaceutical Standard Z14021231 | N |

# 
